# The evolutionary dynamics of DENV 4 genotype I over a 60-year period

**DOI:** 10.1371/journal.pntd.0007592

**Published:** 2019-07-29

**Authors:** Shaowei Sang, Jing Liu-Helmersson, Mikkel B. M. Quam, Hongning Zhou, Xiaofang Guo, Haixia Wu, Qiyong Liu

**Affiliations:** 1 Clinical Epidemiology Unit, Qilu Hospital of Shandong University, Jinan, Shandong, People's Republic of China; 2 Department of Epidemiology and Health Statistics, School of Public Health, Shandong University, Jinan, Shandong, People's Republic of China; 3 Department of Epidemiology and Global Health, Umea University, Umea, Sweden; 4 Yunnan Provincial Center of Arborvirus Research, Yunnan Provincial Key Laboratory of Vector-borne Diseases Control and Research, Yunnan Institute of Parasitic Diseases, Pu'er, Yunnan, People's Republic of China; 5 State Key Laboratory of Infectious Disease Prevention and Control, Collaborative Innovation Center for Diagnosis and Treatment of Infectious Diseases, National Institute for Communicable Disease Control and Prevention, Chinese Center for Disease Control and Prevention, Changping, Beijing, People's Republic of China; University of Oxford, VIET NAM

## Abstract

Dengue virus serotype 4 (DENV 4) has had a relatively low prevalence worldwide for decades; however, likely due to data paucity, no study has investigated the epidemiology and evolutionary dynamics of DENV 4 genotype I (DENV 4-I). This study aims to understand the diversity, epidemiology and dynamics of DENV 4-I. We collected 404 full length DENV4-1 envelope (E) gene sequences from 14 countries using two sources: Yunnan Province in China (15 strains during 2013–2016) and GenBank (489 strains up to 2018-01-11). Conducting phylogenetic and phylogeographical analyses, we estimated the virus spread, population dynamics, and selection pressures using different statistical analysis methods (substitution saturation, likelihood mapping, Bayesian coalescent inference, and maximum likelihood estimation). Our results show that during the last 60 years (1956–2016), DENV 4-I was present in mainland and maritime Southeast Asia, the Indian subcontinent, the southern provinces of China, parts of Brazil and Australia. The recent spread of DENV 4-I likely originated in the Philippines and later spread to Thailand. From Thailand, it spread to adjacent countries and eventually the Indian subcontinent. Apparently diverging around years 1957, 1963, 1976 and 1990, the different Clades (Clade I-V) were defined. The mean overall evolution rate of DENV 4-I was 9.74 (95% HPD: 8.68–10.82) × 10^−4^ nucleotide substitutions/site/year. The most recent common ancestor for DENV 4-I traces back to 1956. While the demographic history of DENV 4-I fluctuated, peaks appeared around 1982 and 2006. While purifying selection dominated the majority of E-gene evolution of DENV 4-I, positive selection characterized Clade III (Vietnam). DENV 4-I evolved in situ in Southeast Asia and the Indian subcontinent. Thailand and Indian acted as the main and secondary virus distribution hubs globally and regionally. Our phylogenetic analysis highlights the need for strengthened regional cooperation on surveillance and sharing of sample sequences to improve global dengue control and cross-border transmission prevention efforts.

## Author summary

Dengue virus (DENV) can be classified into four serotypes, DENV 1, 2, 3 and 4. Although DENV 4 is the first dengue serotype to diverge in phylogenetic analyses of the genus *Flavivirus*, this serotype occurs at a low prevalence worldwide and spreads the least rapidly. Similar to other serotypes, DENV 4 can also cause severe dengue (SD) disease manifestations, such as dengue haemorrhagic fever (DHF) and dengue shock syndrome (DSS). To date, no study has investigated the epidemiology and dynamics of DENV 4 genotype I comprehensively. In this study, we seek to address this gap. Our study shows that the distribution of DENV 4-I is mainly restricted to Southeast Asia and the Indian subcontinent. The most recent spread of DENV 4-I likely originated from Southeast Asia–initially circulating in the Philippines, then Thailand and later on the Indian subcontinent. Viruses evolved in situ in Southeast Asia and the Indian subcontinent, respectively. Although DENV 4-I occasionally spread elsewhere, this genotype did not become widely established. The overall evolution rate of DENV 4-I was comparable with that of DENV 2–4. The nucleotide sequences indicates that the demographic history of DENV 4-I fluctuated with peaks apparent during parts of the 1980s and 2000s. Although a weak positive selection existed in Clade III -predominately in Vietnam, purifying selection dominated the E-gene evolution of DENV 4-I.

## Introduction

Dengue is a mosquito-borne viral infectious disease. Although the geographical origin of dengue is still under some debate, the recent global expansion has been attributed to environmental changes, unprecedented population growth, uncontrolled urbanization, spread of the mosquito vectors, and host population movements [1]. Currently, dengue is endemic in more than 100 countries in much of the globe’s tropical and subtropical areas, being reported predominantly in Southeast Asia, the Americas, and the Western Pacific, and less frequently in Africa and Eastern Mediterranean WHO regions [2]. The prevalence of dengue has increased 74.7% between 2006 and 2016 [3]. While dengue infections are most often asymptomatic, a recent study has estimated a global total of 58.4 (95% CI: 24–122) million symptomatic dengue cases occur annually costing some US$8.9 (95% CI: 3.7–19.7) billion [4].

The four antigenically distant serotypes comprising dengue virus are categorized as DENV 1, -2, -3, and -4. Each serotype is classified into different genotypes based on complete E gene sequences [5]. The four serotypes were first identified at different times and locations, and diffused globally at different rates. Although clearly circulating before isolation techniques enabled the viruses’ discovery and characterization, DENV 1 was first reported in 1943 in French Polynesia and Japan, DENV 2 in 1944 in Papua New Guinea and Indonesia, DENV 3 and DENV 4 both in 1953 in the Philippines and Thailand [6]. A study mapping the global spread of DENV 1–4 over the 70-year history (1943–2013), indicated DENV 1 was reported most often, followed by DENV 2, DENV 3, and DENV 4 [7]. Although, DENV 4 was the first dengue serotype to diverge in phylogenetic analyses of the genus *Flav*ivirus [8], it spread the slowest geographically [7]. Similar to other serotypes, DENV 4 can also cause severe dengue including dengue haemorrhagic fever (DHF). An epidemiological study in Thailand from 1973 to 1999 revealed that despite the proportionately lower prevalence of DENV 4, it was responsible for 10% of all DHF cases in Children [9].

Among DENV 4, there are four genotypes (I, II, III and Sylvatic). The dengue cases from DENV 4 genotype I (DENV 4-I) have increased in recent years. In 2013, a large DENV outbreak occurred in central Vietnam with a total of 204,661 clinical cases reported, of which 48.9% were DENV 4-I cases [10]. The same year, DENV 1-I, DENV 2-I and DENV 4-I caused a large outbreak with 20,255 cases including 84 deaths in Myanmar [11]. In Sri Lanka, the dominant virus in the 2012 epidemic was DENV 1, but DENV 4-I infections were also commonly observed [12]. While all four serotypes have been detected, a 2015 study showed that dengue in China remains primarily an imported disease with DENV 1 most frequently found in samples [13]. However, since 2013 several strains of DENV 4-I have been detected in Yunnan Province, China. Among the recorded DENV 4-I strains, both imported and autochthonous cases were found.

The dengue viruses DENV 1–4 are typically prevalent in tropical and subtropical regions globally. However, the spatial distribution of different genotypes is not uniform, e.g., some genotypes exist only in specific parts of Asia and others are more cosmopolitan. While the distinct distribution patterns of different genotypes remain enigmatic, mapping of the genotypes’ distribution can generate hypotheses on their spatial pattern and support policies on dengue prevention and control effort. In the past, efforts have been made to infer the dispersal of DENV 1–4 and to elucidate the evolution of their diffusion patterns [14–17]. However, so far no studies investigated globally the spatial distribution of the single genotype, DENV 4-I, its diversity, and its temporal evolution. This may be due to the limited number of recorded cases and the distribution of DENV 4-I worldwide.

In this study, we used available GenBank data in addition to Chinese data sources to make the first attempt to more comprehensively understand the spatial and evolutionary patterns of DENV 4-I. Leveraging full envelope gene sequences in our analysis, we sought to investigate the origin and spreading routes of this less-studied, rare but deadly virus, in order to contribute important information for future dengue prevention and control efforts around the globe.

## Methods

### Ethics statement

Ethical approval for the study was obtained from the Chinese Center for Disease Control and Prevention Ethical Committee (No. 201214).

### Data collection

Dengue viruses detected in Yunnan Province were recovered from serum samples of suspected dengue patients visiting hospital from 2013 to 2016. The envelope (E) genes of isolates were sequenced as described previously [18]. These have been assigned GenBank accession numbers (HM893690-HM893699, MG601754, KJ470764, KJ470765, KX262920, KX262923). The sequences of Yunnan were compared with published sequences by using the nucleotide blast program in the NCBI. All the sequences of human DENV 4-I with full length E-gene (1,485 nucleotides) were downloaded with the accession number, collection date and country/region (as of January 11^th^, 2018). All the sequences were aligned using MAFFT [19]. We chose only one sequence to represent sequences with 100% matching identity by location and time. Recombinants detected based on the analyses of RDP3 program [20] were also excluded. Ultimately, 404 sequences of DENV 4-I obtained from 14 countries were included for analyses in this study (S1 Table).

### Substitution saturation analysis and likelihood mapping analysis

The phylogenetic signal of the aligned DENV 4-I was evaluated by plotting the observed number of transitions and transversions against genetic distance for the n (n-1)/2 pairwise comparisons in an alignment of n taxa using DAMBE [21]. It is expected that transitions and transversions increase linearly with the genetic distance, with transitions being more frequent than transversions. In the Supplementary Materials, S1 Fig shows that no substitution saturation was detected, indicating phylogeny reconstruction was appropriate. In likelihood mapping analysis, groups of four sequences (quartets) were evaluated using the maximum likelihood approach. For each quartet, the three possible unrooted tree topologies were weighted. The likelihood weights were then plotted into a triangular surface. The fully resolved tree topologies were plotted in the three corners, which indicated the presence of a tree-like phylogenetic signal, and the unresolved quartets, indicating a star-like signal were shown in the central region of the triangle [22]. Likelihood mapping was performed using the TREE-PUZZLE program [23], by analyzing 10,000 random quartets. S1 Fig showed tree-like area accounted for 96.4%, which further suggested that the data were reliable for phylogenetic inference.

### Phylogenetic and phylogeographical analyses

Rates of nucleotide substitution per site per year and time to The Most Recent Common Ancestor (TMRCA) were estimated using Bayesian Markov Chain Monte Carlo (MCMC) and implemented using the BEAST v1.8.2 software package [24]. The best-fit model of nucleotide substitutions was determined using Bayesian Information Criteria (BIC) as implemented in jModelTest [25]. The calibration point was the “year” that each strain was isolated. Statistical simulations were performed under strict or relaxed (uncorrelated exponential and lognormal) clock model, with the Bayesian Skyride Coalescent Tree Prior [26]. To determine the best-fit combination, we have applied Posterior-simulation Akaike Information Criterion through MCMC (AICM) [27], Bayes Factor (BF) [28], Harmonic Mean (HM) [29], and Path Sampling (PS) and Stepping-stone Sampling (SS) [30] model selection methods. The results showed that the best fitting model was the combination of uncorrelated relaxed exponential clock model and the Bayesian Skyride Coalescent model (S2 Table). Statistical uncertainties in parameter values were given by the 95% Highest Probability Density (HPD) intervals. All chains were run sufficiently long to achieve convergence (the effective sample size of continuous parameters greater than 200) after burn-in, as checked using TRACER v1.5 (http://tree.bio.ed.ac.uk/software/tracer/). The programs TreeAnnotator v1.8.2 in the BEAST v1.8.2 software package and Figtree (http://tree.bio.ed.ac.uk/software/Figtree/) were used to summarize the posterior tree distribution and to visualize the annotated Maximum Clade Credibility (MCC) tree, respectively. Based on the MCC tree, we identified five Clades using visual judgement and comparison among all the countries/regions that reported DENV 4-I. Using the definition of a minimum of three sequences of monophyletic origin, DENV 4-I were labelled with Clade I to V (the largest five) where every Clade included as many strains as possible.

The spatial diffusion of DENV 4-I was estimated using the Bayesian Markov chain Monte Carlo (MCMC) statistical framework implemented in the BEAST v1.8.2 package. The phylogeographical diffusion process was identified using the Bayesian Stochastic Variable Search Selection (BSVSS). Effective population size dynamics were estimated using the Bayesian Skyride Coalescent statistical approach.

**Fig 1:**
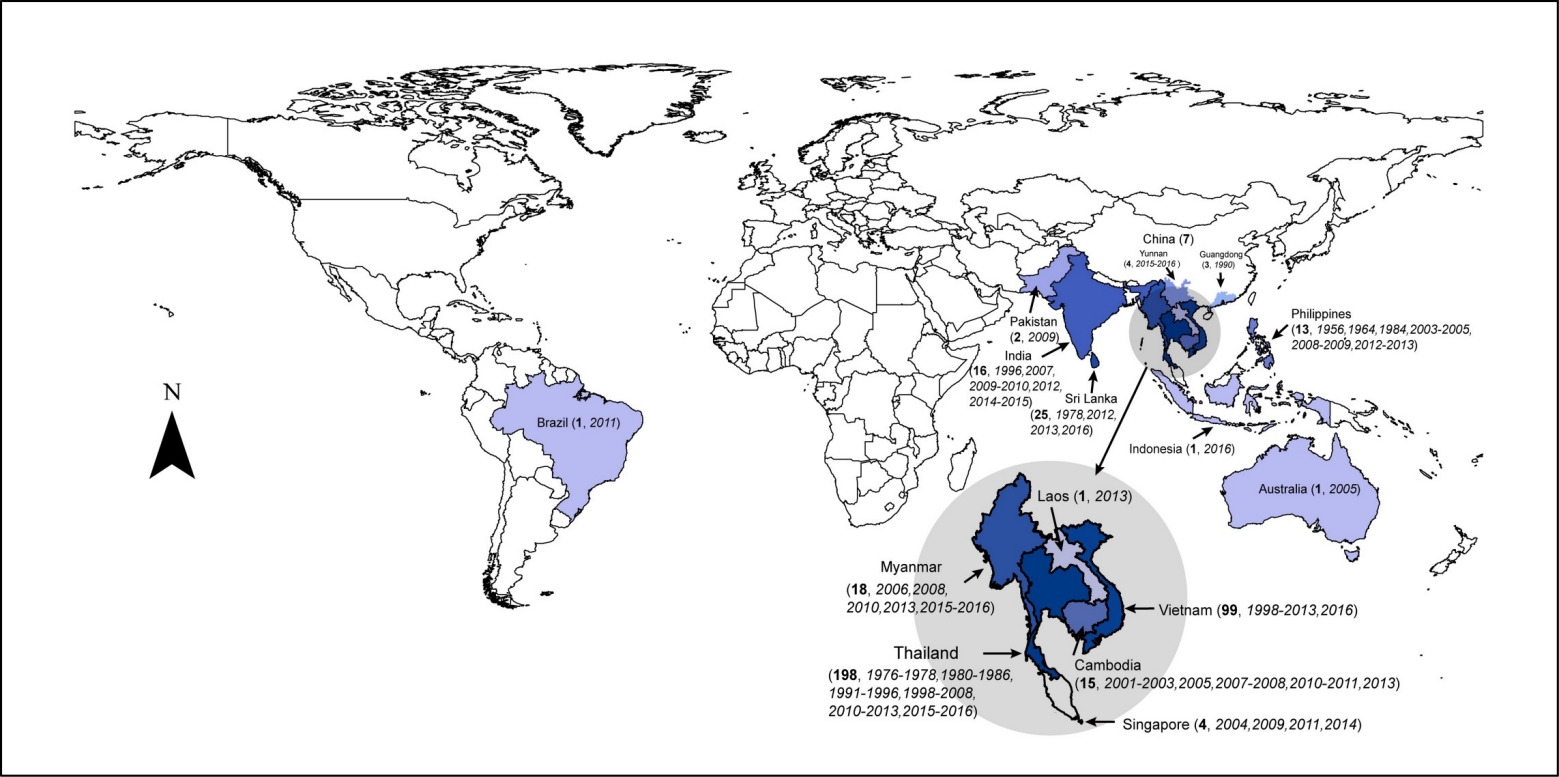
The global distribution of DENV 4-I. The number of unique strains (in bold, < 100% similarity in the same year) and the years of recorded transmission (in italic) in each location are shown in parenthesis after each location name.

Open source data from http://tapiquen-sig.jimdo.com (Carlos Efraín Porto Tapiquén. Orogénesis Soluciones Geográficas. Porlamar, Venezuela, 2015) were used in this study for the results shown in Figs 1 & 4 with help of ArcGIS 10.2 and Adobe Illustrator.

### Estimating selection pressure

We used a variety of computational methods to explore the selection pressures. A Maximum Likelihood (ML) method was used to examine selection pressures [31]. In the analysis, the non-synonymous to synonymous rate ratio (*ω = d*_*N*_*/d*_*S*_) was determined codon-by-codon using various models of codon substitution. These models differ in how *ω* ratios are allowed to vary along the sequence. Four models of codon substitution were conducted in the study: M1a (*ω* < = 1; nearly neutral), M2a (*ω* < = 1 and *ω* > 1; positive selection), M7 (beta; a discrete distribution with 10 site classes to model values of *ω* between 0 and 1) and M8 (beta and *ω* > 1). M1a is nested with M2a, and M7 is nested with M8. Models that are nested are compared statistically using a Likelihood Ratio Test (LRT). Positive selection can be inferred when a group of codons with a *ω* ratio > 1 is identified and the likelihood of the codon substitution model in question is significantly higher (*p* < 0.05) than that of a nested model that does not take positive selection into account. Lastly, using Bayes Empirical Bayes (BEB) methods, posterior probabilities were calculated to identify sites under positive selection (posterior Bayesian probability (*P*p) > 95%). All the analyses were performed by using CODEML from the PAML v4.9 package [32].

Evolution rate, effective population size dynamics, divergence time and selection pressure were estimated based on two different types of datasets: (i) all sequences of DENV 4-I and (ii) those from different Clades. The spatial diffusion was estimated based on all sequences of DENV 4-I. In order to minimize oversampling of Thailand and Vietnam during the spatial diffusion analysis, we down-sampled dataset for sensitive analysis. The down-sampled dataset included 50 sequences at random, from each Thailand and Vietnam and all the available sequenced strains from other countries/regions, therefore making the sample size 207 sequences.

## Results

### Epidemiological characteristics of DENV 4-I in the world

Fig 1 shows that DENV 4-I were detected in Mainland Southeast Asia and the adjacent provinces of China, Maritime Southeast Asia, the Indian subcontinent, Brazil and Australia. Specifically, the recorded samples revealed that DEVN 4-I was mainly observed in Mainland Southeast Asia, especially Thailand and Vietnam. Collection of DENV 4-I covered a period of 60 years. The first strain of DENV 4-I was detected in 1956 in the Philippines, where it transmitted exclusively for some 20 years, according to known reporting and sequencing records. Over the two decades following 1976, most detected strains of DENV 4-I were found to be circulating in Thailand, while a few strains were discovered in other four countries. Detected in a total of 14 countries, DENV 4-I continued to diffuse to more areas around the globe between 1996 and 2016.

**Fig 2:**
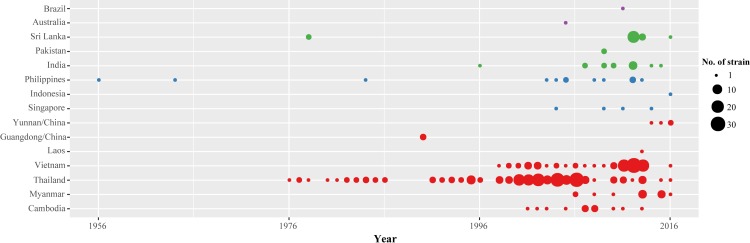
The temporal distribution of DENV 4-I by location. The red color dots represents the strains detected in mainland Southeast Asia and its adjacent provinces of China; similarly, blue depicts those found in maritime Southeast Asia; green those from the Indian subcontinent; and purple those few strains found in circulation elsewhere (Brazil and Australia). The diameter size of the circles corresponds to the number unique strains of DENV 4-I in a given year in a given location.

Fig 2 shows the evolution and spread of DENV 4-I over time. During the last 60 years, great geographical and genetic diversity has occurred. This is especially prominent during the last two decades after DENV 4-I became more prevalent as shown in the genetic record.

### Phylogenetic and phylogeographical analyses of DENV 4-I

**Fig 3:**
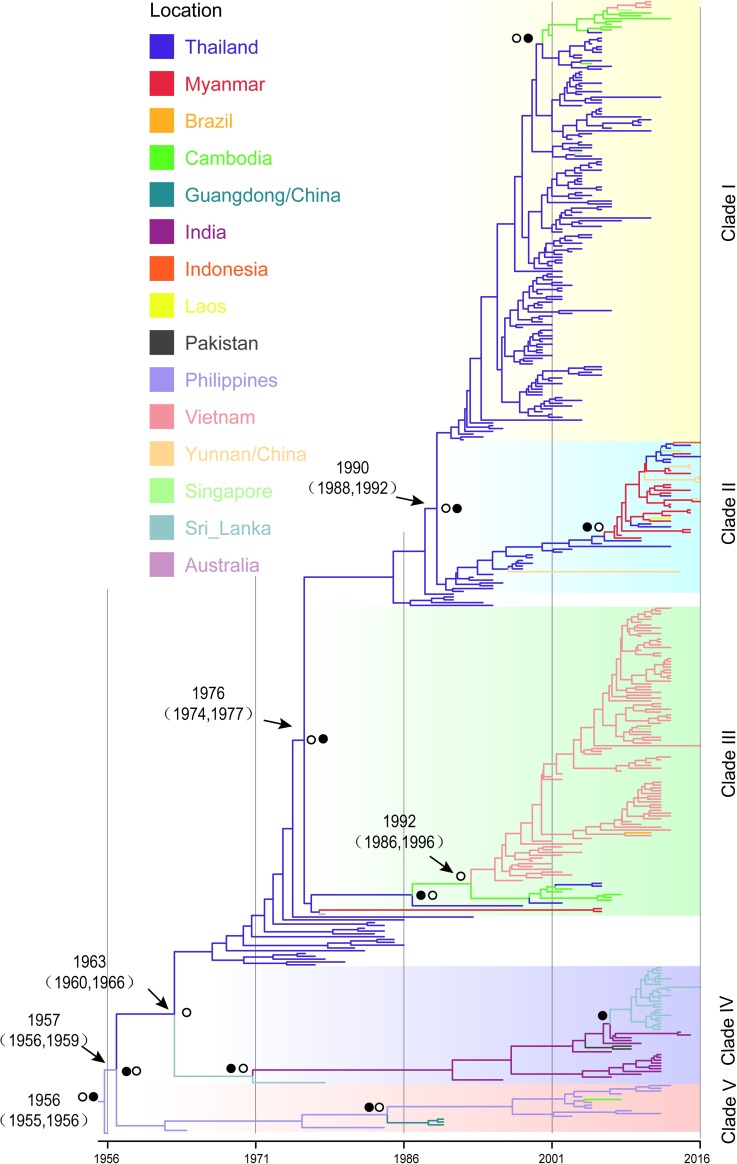
The Maximum Clade Credibility (MCC) tree summarized for DENV 4-I. The colors of the branches correspond to their probable geographic location (see the legend). Circles and black dots indicated posterior probability support and ancestral location state probability of main node > = 0.85. For the key nodes, the median estimated divergence time and respective 95% HPD intervals were shown.

Fig 3 shows the Maximum Clade Credibility (MCC) tree. It indicates that the recent spread of DENV 4-I most likely originated from the Philippines with 0.98 posterior location probability. Viruses evolved in the Philippines and then spread across the sea to Thailand, Cambodia, Australia and China at different times. Thailand played the dominate role in spreading the viruses, gradually spreading virus to the Indian subcontinent, Myanmar, Cambodia, Singapore, Indonesia and China. These viruses diverged around 1957, 1963, 1976 and 1990, and shaped different Clades (Clade I to V). Since the introduction from Thailand, DENV 4-I has evolved in the Indian subcontinent (Clade IV), Myanmar (Clade II) and Cambodia (Clade I and III), respectively. Strains obtained in Vietnam correspond to Clade III, which evolved notably in situ for three decades after introduction from Cambodia. In the Indian subcontinent (Clade IV), dengue virus first apparently arrived in Sri Lanka in the 1960s from Thailand and then spread onto India in the early 1970s. India then became an epicenter for transmission and spread virus to Pakistan and back to Sri Lanka in the 2000s.

**Fig 4:**
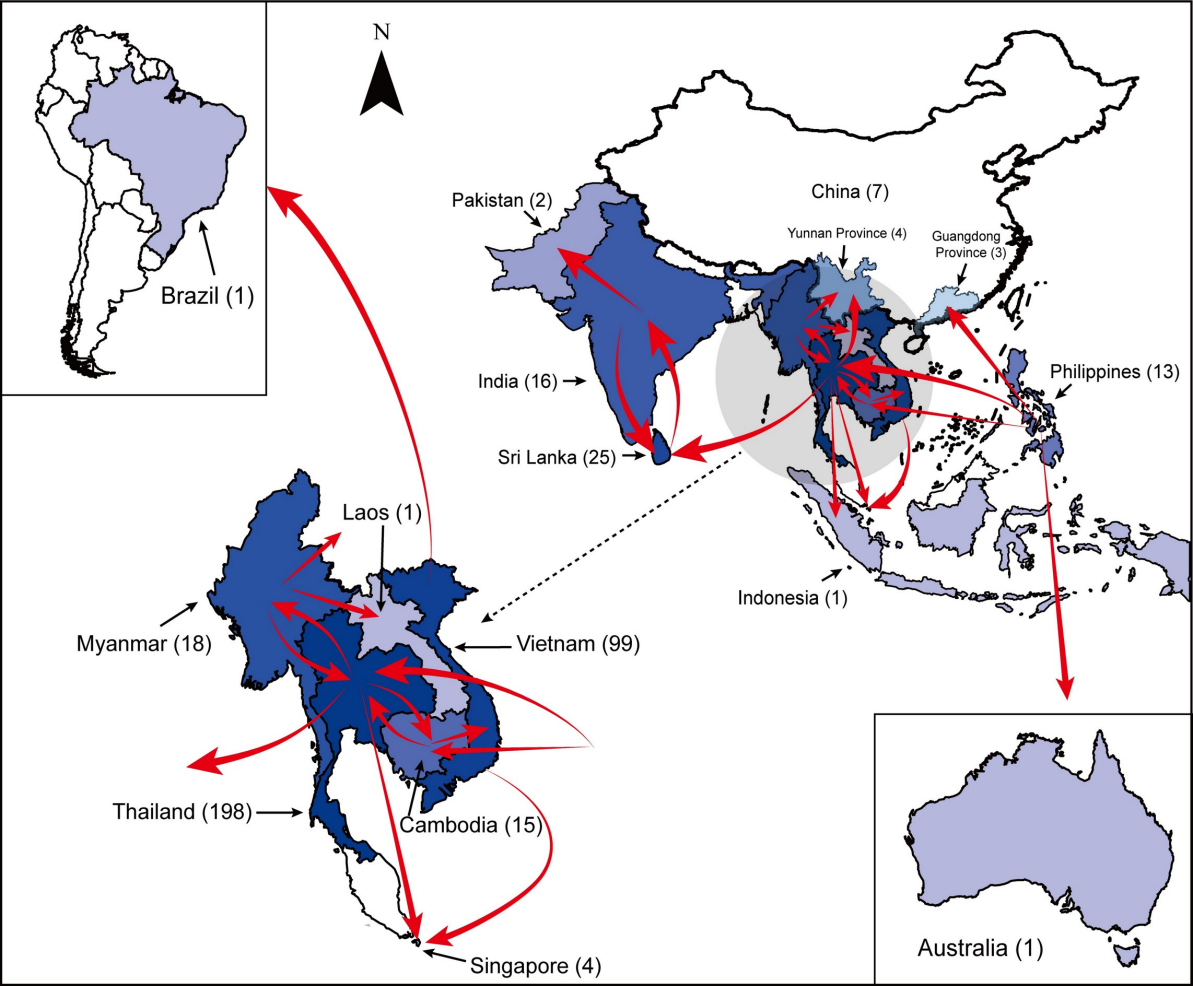
The global dissemination pathways of DENV 4-I. Driven by the phylogeographical analysis of all included strains, red arrows indicate the probable direction of trans-border expansion of DENV4-1. The number of unique strains (<100% similarity in the same year) in each location is shown in parenthesis after each location name.

Fig 4 shows the detailed spatial diffusion of DENV 4-I as summarized from the MCC tree. The result of the down-sampled dataset showed that the phylogenetic topology and spatial spreading patterns were equivalent with those from full dataset (S2 Fig).

### Demographic history of DENV 4-I and Clades evolution

**Fig 5:**
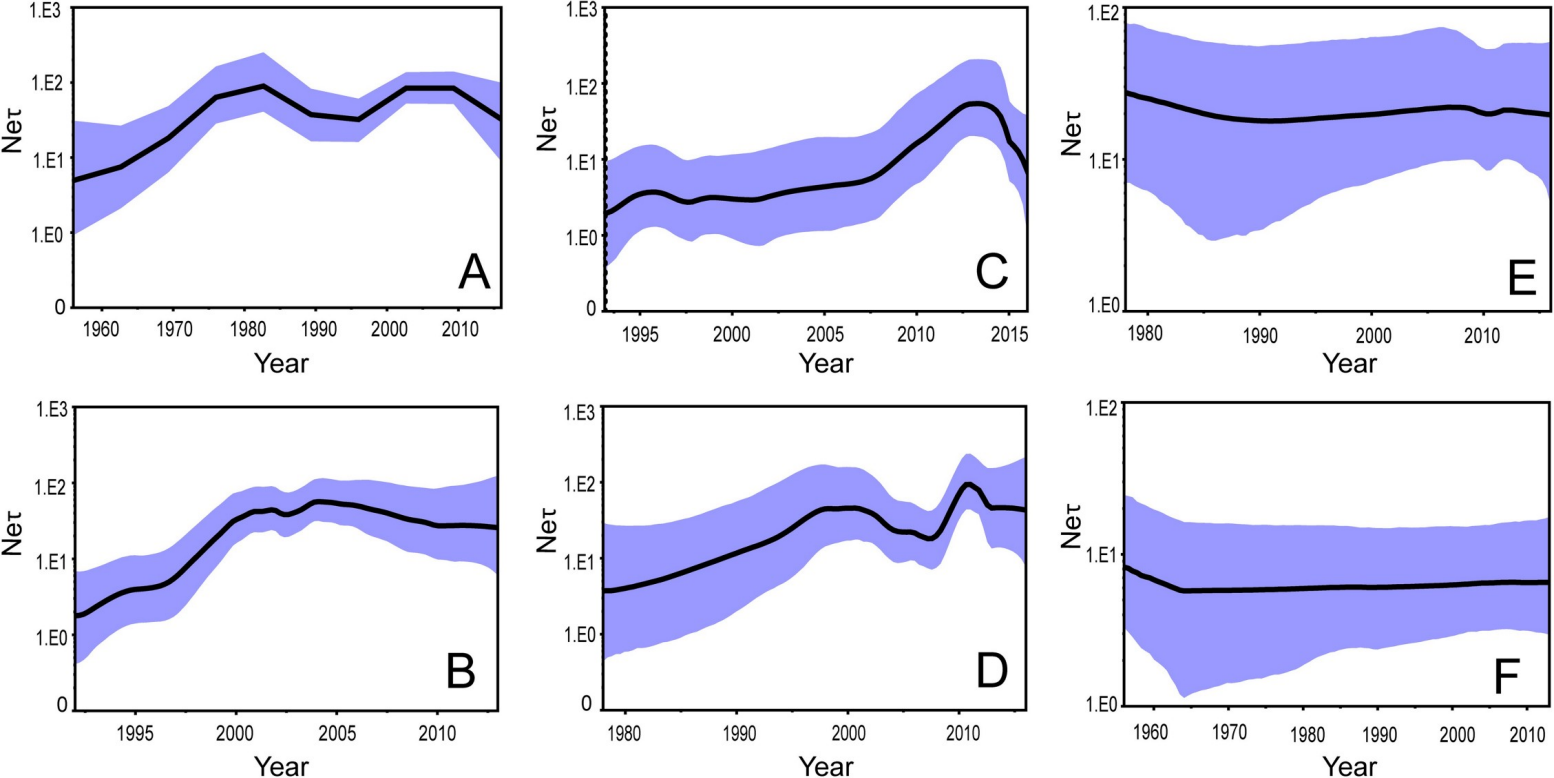
The demographical history of total DENV 4-I (A) and Clade I to V (B-F). The black line represents the median posterior value, and the blue area represents the 95% HPD intervals. The x-axis corresponds to time (years), while the y-axis is the product of the effective population size and the generation in years (N_e_τ).

Fig 5A illustrates the demographic history of DENV 4-I. A fluctuation was observed over the 60 years with an approximately “M” shape. The two highest plateaus were around 1982 and 2006 with a width about 6-years, while the lowest point was around 1996. Analysis of the Clade I dataset revealed, as Fig 5B exhibits, that the effective population size increased approximately linearly from 1992 to 2004, and then decreased slowly from 2004 to 2013. The effective population size of Clade II, as Fig 5C conveys, increased slowly for the first two decades and then much more sharply (2007–2013), before a rapid recent decrease (2013–2016). Data from the Clade III in Fig 5D shows that there were two peaks (~2000 and ~2011) over nearly four decades of demographic analysis. The effective population size of Clade IV over the same period decreased slowly and then stayed constant with the inflexion lying around 1990, as Fig 5E depicts. In Fig 5F, one can see the long demographic history pattern of Clade V (1956–2013) is mirrored by Clade IV, but with the inflexion point much earlier (~1965).

### Evolution rate

Table 1 shows the evolution rate and the divergence time of the different Clades’ datasets. The overall evolution rate was 9.74 × 10^−4^ (95% HPD: 8.68 × 10^−4^–10.82 × 10^−4^) nucleotide substitutions/site/year and TMRCA of DENV 4-I was 1956 (95% HPD: 1955–1956) (year). The mean evolution rate of different Clades was comparable, with the smallest being 8.66 × 10^−4^ nucleotide substitutions/site/year in Clade V and the largest being 11.3 × 10^−4^ nucleotide substitutions/site/year in Clade II. TMRCA of Clade I to V was 1991, 1990, 1977, 1971 and 1962, respectively.

**Table 1:** Nucleotide substitution rates and divergence times for the E-gene from different datasets.

Dataset	Nucleotide substitution rate (x10^-4^ substitution/site/year) (95% HPD)	TMRCA (95% HPD, year)
DENV 4-I	9.74 (8.68, 10.82)	1956 (1955, 1956)
Clade I	9.42 (7.78, 11.04)	1991 (1989, 1992)
Clade II	11.3 (8.34, 14.44)	1990 (1988, 1992)
Clade III	8.95 (6.97, 11.06)	1977 (1975, 1978)
Clade IV	9.19 (4.66, 13.46)	1971 (1964, 1976)
Clade V	8.66 (5.90, 11.57)	1962 (1958, 1964)

### Selective pressure

In the Supplementary Material, S3 Table shows the summary of positive selection analysis performed on different datasets. M1a *vs* M2a test and M7 *vs* M8 test indicated consistently that there was no positive selection across overall DENV 4-I, Clade I, II, IV and V datasets. In Clade III dataset, no positive selection was indicated by M1a *vs* M2a test, however, M7 *vs* M8 test inferred a weak positive selection (5.6% of codons with *ω* = 1.193) with amino acid site 95 *P*p > 0.95.

## Discussion

In this study, using the most comprehensive and largest dataset(s) available we mapped the spatial distribution and 60-years evolution of one genotype from one of the four serotypes of dengue virus, DENV 4-I. We further separated this large dataset into different Clades and analyzed the evolutionary dynamics of Clades separately. Our study shows that the spatial distribution of DENV 4-I is mainly restricted to Southeast Asia and the Indian subcontinent. The recent spread of DENV 4-I likely originated from Southeast Asia–namely the Philippines, from where it then spread all the way to the Indian subcontinent, Australia and Latin America. DENV 4-I evolved in situ in Southeast Asia and on the Indian subcontinent. Although DENV 4-I cases occasionally were found elsewhere, this genotype didn’t undergo in situ evolution and largely failed to establish. We found that mainland Southeast Asia, specifically Thailand, was at the center of the global spread of the viruses, which in time contributed to the observed diversity.

Although Thailand’s strains dominated the records, the Philippines’ strains were at the basal location of the phylogeny. The analyzed diverge time (95% HPD: 1955–1956) is consistent with the timing of DENV 4-I virus being first detected in the Philippines. Therefore, we propose that global spread of DENV 4-I originated very likely from the Philippines. From the Philippines, DENV 4-I spread to Thailand and then on to nearby countries including Sri Lanka. On this point our findings clash with one previous study’s results indicating that DENV 4-I originated from Thailand, from where it spread to the neighboring countries of the Philippines and Sri Lanka [17].

We found that the strains of DENV 4-I—monophyletic Clade IV, detected in the Indian subcontinent including Sri Lanka, India and Pakistan, probably originated in Thailand, then evolved in situ, but as of 2016, had not yet spread outside of the Indian subcontinent. Dengue is endemic in the Indian subcontinent and four serotypes have been co-circulating, with DENV 2 and DENV 3 dominating [33]. However, the distribution and dissemination patterns of the different genotypes are not uniform. A study showed that the DENV 3 genotype III (DENV 3-III) was spread from the Indian subcontinent to East Africa first and then from there to Latin America [34]. Another study on DENV 2 showed that the cosmopolitan genotype was spread from the Indian subcontinent directly to Latin America [14]. In addition to the Indian subcontinent, dengue is also endemic in Brazil. Even in two separate regions of the world where dengue is endemic, the specific evolutionary drivers of a given serotype and genotype may differ.

DENV4-I was introduced to Brazil in 2011. Our study indicated that the strain was probably imported from mainland Southeast Asia, which was consistent with the analysis conducted in a previous study [35]. Curiously, DENV 4-I apparently disappeared quickly from Brazil after the introduction. This is different from the expansion/establishment patterns of DENV 4-II, which has become established in the Caribbean since early 1980s after being introduced from Southeast Asia [36]. Human movement is known to play a significant role in contributing to the virus distribution at large spatial scales (e.g., national, international) [37], because of the limited range of mosquitos’ flying distance. However, human movement alone cannot explain the different expansion pathways and establishment of new genotypes, for example, in the DENV 4-I dynamics on the Indian subcontinent and Brazil even after controlling for the climate suitability and mosquito abundance. These studies show that the spread of dengue virus is not merely shaped by human movement. Human movement is a necessary but not sufficient condition. Different pathways of genotypes spread may be associated with the strain virulence, human population immunity, previous exposure to other dengue viruses, in addition to the volume and timing of human population movements, the local environment, and numerous other factors.

Our study on evolution rate showed that the substitution rate of DENV 4-I is 9.74 (95% HPD: 8.68–10.82) × 10^−4^ nucleotide substitutions/site/year. This is comparable with the value found by Klungthong *et al*. [38] (10.72 (95% HPD: 8.41–13.11) × 10^−4^ nucleotide substitutions/site/year), but is approximately twice that of the one estimated by Twiddy *et al*. (5.42 × 10^−4^ substitutions/site/year) using an admittedly much smaller dataset [39]. The range of mean evolution rate from DENV 2–4 is found to be (8.3–10.7) × 10^−4^ nucleotide substitutions/site/year [16], which included Asian I and Asian-American genotype of DENV 2, genotype I-III of DENV 3, DENV 4-II. Although DENV 4-I was shown to evolve with a relatively large substitution rate comparing to some other genotypes [16], there is no significant rate differences among the different dengue serotypes. Among the five Clades of DENV 4-I, we found that the highest evolution rate was among Clade II strains (Table 1). The MCC tree (Fig 3) indicates that the strains of Clade II spread rapidly in mainland Southeast Asia. The rapid spread and the subsequent replication in a large susceptible population of hosts could account for the higher rate of evolution, when compared to the other Clades in DENV 4-I having relatively smaller pools of susceptible hosts. We found no evidence of positive selection in most of the Clades of DENV 4-I, although there was evidence for weak positive selection in Clade III. Some studies have suggested purifying selection [38], where majority of amino acid changes within infected hosts, are deleterious in the long run and are eventually removed from the population [40]. This purifying selection induces the distinct ladder-like phylogeny. Our study confirms this purifying selection process as the predominant evolutionary force acting on DENV 4-I.

Our study also showed that DENV 4-I experienced a fluctuating demographic pattern although occurring in a low prevalence compared to other serotypes. The fluctuation of effective infected population size might be shaped by human population immunity/susceptibility. DENV 4-I was first detected in the Philippines and spread to Thailand and Sri Lanka before 1980, where humans were lacking previous exposure and thus immunity. The effective infected population size decreased once immunity was built up as result of previous exposure. DENV 4-I viruses spread onto Vietnam around 1992 (95% HPD: 1986–1996) and evolved in situ, which might have induced the secondary increase. This observation agrees with the study by Villabona-Arenas *et al*. using the dataset from 1956 to 2008. They indicated that the effective population size was estimated to be the largest at two time periods around 1982 and 2005 [17], which is similar to our result shown in Fig 5A using a larger dataset covering a longer period.

Dengue is endemic in Vietnam. Most of the DENV 4-I strains detected in Vietnam shaped the Clade III, as the in situ evolution occurred since DENV 4-I started circulating in Vietnam in 1998. It did not dominate during dengue epidemics until 2013 when a large outbreak was occurred having 204,661 clinical cases with nearly 50% of them having DENV 4-I in central Vietnam [10]. This occurrence coincided with the demographic history of Clade III. Lacking immunity to DENV 4-I among the Vietnamese human population could be a reason to cause second infection and therefore severe dengue which resulted in being more likely reported. Amino acid 95 under positive selection could also account for this large outbreak. The E protein ectodomain can be divided into three structural domains designated domain I-III. Domain II contains the fusion loop (residue 98–111), which interacts with the host endosomal membrane, leading to virus-mediated membrane fusion and allowing the newly infecting virus to initiate the cellular replication cycle. As residue 95 is located three residues downstream of the fusion loop, it is likely to indirectly affect the process of membrane fusion. The Vietnam strains experiencing positive selection pressure did not spread outside of Vietnam based on the available data and our findings. Enhanced surveillance to these strains could be very helpful to aid in understanding and controlling this potentially devastating virus strain.

Although our study represents the most comprehensive study on the evolutionary dynamics of DENV 4-I using the largest dataset available to date, the results should be interpreted cautiously given various limitations. For example, itself, reporting of DENV, especially in the genetic record, is a source of many types of potential bias, of particular concern in locations with limited resources for virologic diagnostic and reporting capacity. While our dataset represents an opportunistic, but highly useful sample of occurrence, it should be noted that we worked with a limited subset of data, rather than a complete record of global DENV 4-I transmission dynamics. To account for sampling bias, in this study we constructed and analyzed a down-sampled dataset for sensitive analysis, finding an equivalent result to that which was obtained with the full dataset. Nevertheless, the quality of this type of study will be increased greatly with an enhanced global dengue surveillance, greater access to next generation diagnostic and sequencing tools, and improved data sharing systems. Our current study can be described as exploratory research, as this is the first time that the geographic spread and evolutionary dynamics of the DENV 4-I was mapped out and analyzed based on a large dataset. Given that other dengue research in this area is sparse and rarely conducts genotype-specific analysis, our targeted focus on just one genotype of DENV 4 makes comparison difficult. We hope that our study can stimulate further research studies in this area so that in the future researchers can compare different genotypes and understand better the similarities and differences among them. Our mapping of the genotypes’ distribution pattern may help to generate hypotheses on the specific mechanisms mediating the spread of DENV 4-I. This understanding is potentially of great utility in the generation of health policies and practices on dengue prevention and control.

### Conclusion

In this study, we have investigated the global patterns of DENV 4-I dissemination—its spatial and temporal distribution. This is the most extensive molecular epidemiological study of DENV 4 genotype I to date to our knowledge. Our results indicate that recent spread of DENV 4-I originated in maritime Southeast Asia, probably from the Philippines, from where it spread to mainland Southeast Asia, and then on to the Indian subcontinent. Thailand acted as a distribution hub for spreading the virus regionally and globally. Within the India subcontinent, India was the distribution center for spreading the virus. We found that there is no uniform spreading pattern among genotypes. In addition, purifying selection was still the dominant acting force on E gene to shape the evolution, but weak positive selection existed in dengue viruses detected in Vietnam.

This work is a first step towards increased understanding of the underlying mechanisms governing the spread of DENV 4-I virus. Our study suggests that surveillance could be enhanced to better leverage next generation sequencing for informing dengue control practices. Regional cooperation should be strengthened to determine and communicate information on the genotype-specific spreading pathways, to explore the related underlying mechanisms, and ultimately to better coordinate dengue control efforts globally.

## Supporting information

S1 Fig Results of substitution saturation analysis (A) and likelihood mapping analysis (B).
(TIF)

S2 Fig Maximum Clade Credibility (MCC) tree summarized for down-sampled dataset of DENV 4-I. The colors of the branches corresponded to their probable geographic location (see the legend). The number was the ancestral location probability of key node.
(TIF)

S1 Table DENV4 genotype I. Strains from GenBank were included in the analyses.
(XLSX)

S2 Table Model selection based on marginal likelihood estimates. (XLSX)

S3 Table Parameters estimated from selection pressure analyses. Here positive selection was detected based on different datasets.
(XLSX)
