# Supplementary figures and images for: The evolutionary dynamics of DENV 4 genotype I over a 60-year period
Source: PLoS Negl Trop Dis. 2019 Jul 29;13(7):e0007592. doi: 10.1371/journal.pntd.0007592 (PMC6663010; doi:10.1371/journal.pntd.0007592)

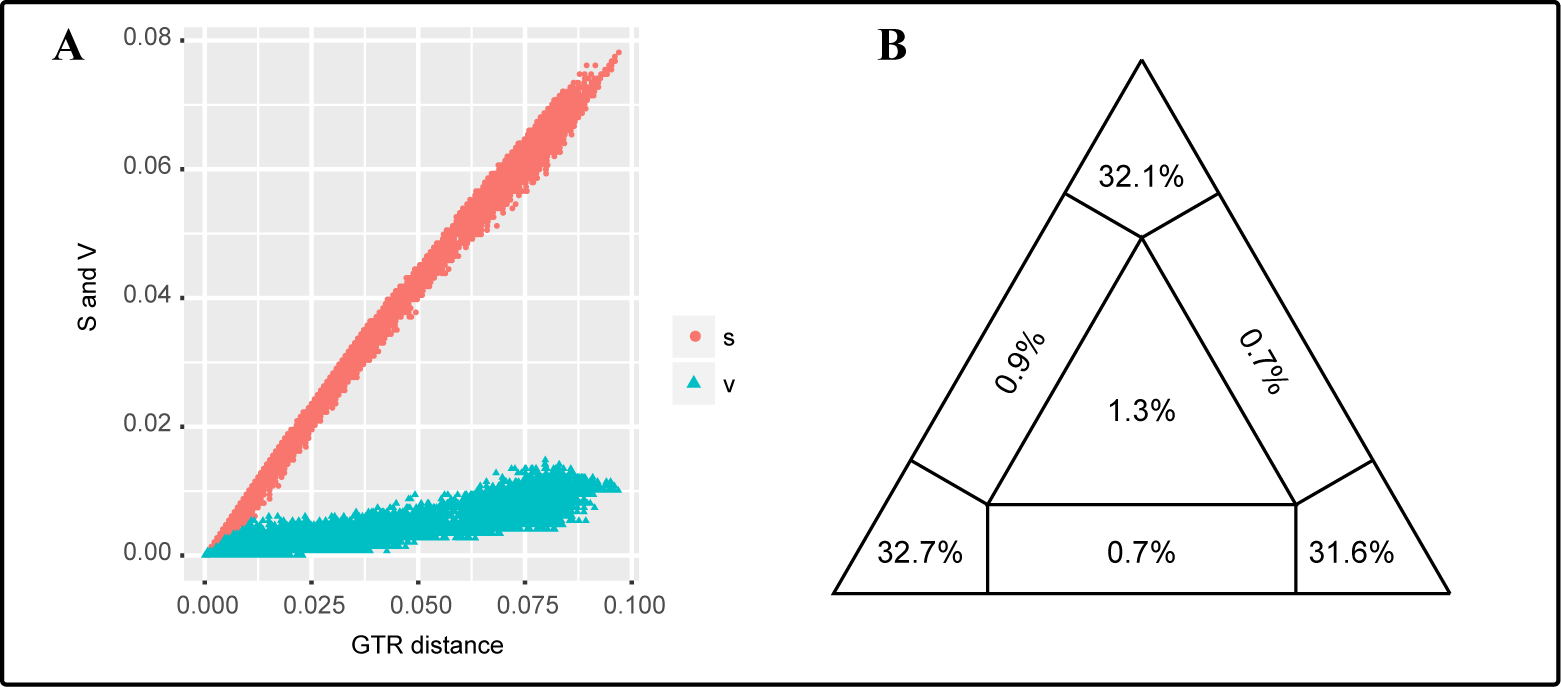

Supplement: S1 Fig — Results of substitution saturation analysis (A) and likelihood mapping analysis (B). (TIF) [file pntd.0007592.s001.tif]

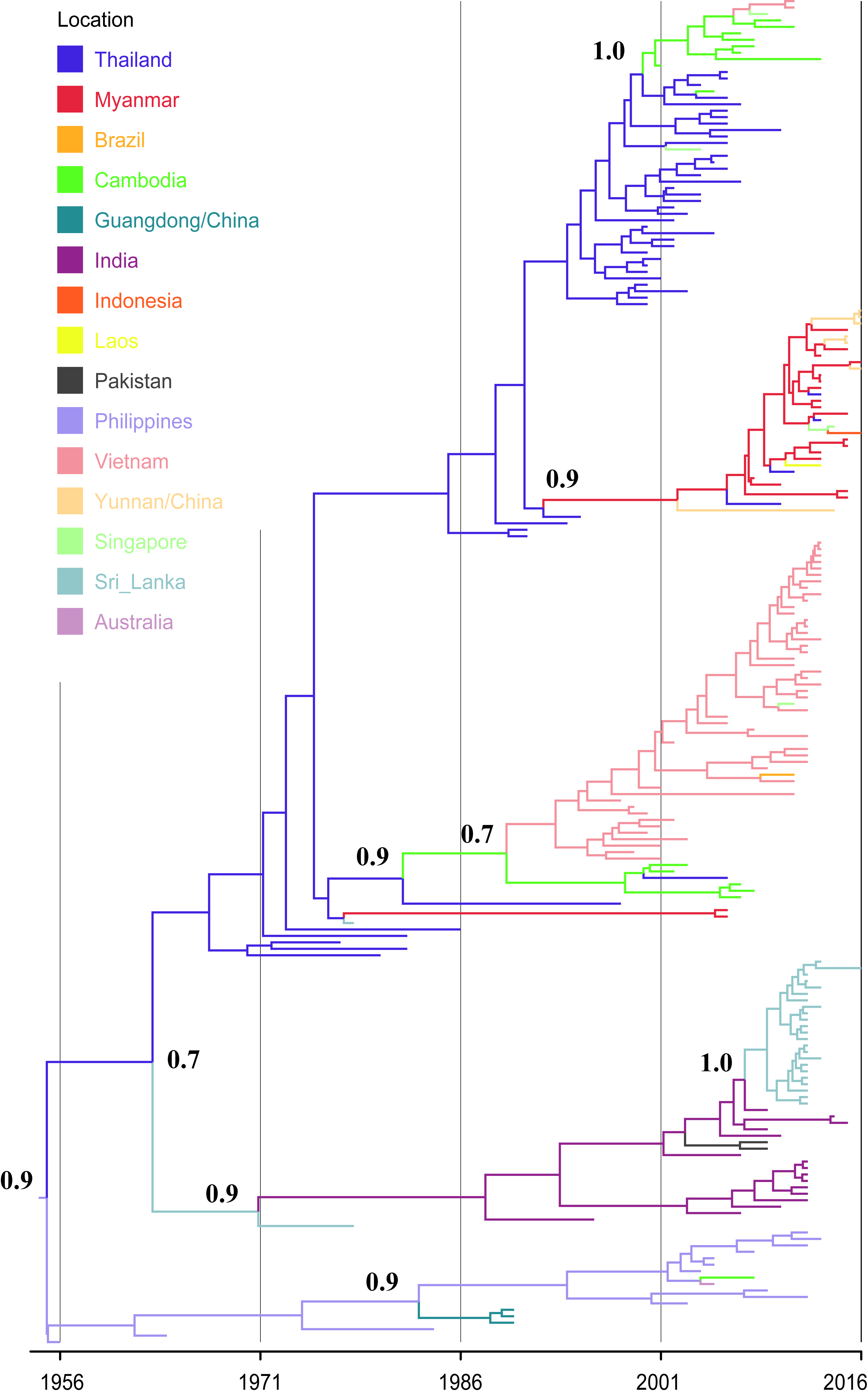

Supplement: S2 Fig — The colors of the branches corresponded to their probable geographic location (see the legend). The number was the ancestral location probability of key node. (TIF) [file pntd.0007592.s002.tif]
